# Supplementary material for: The impact of Neglected Tropical Diseases (NTDs) on health and wellbeing in sub-Saharan Africa (SSA): A case study of Kenya
Source: PLoS Negl Trop Dis. 2021 Feb 11;15(2):e0009131. doi: 10.1371/journal.pntd.0009131 (PMC7904142; doi:10.1371/journal.pntd.0009131)
Supplement: Attachment — Submitted filename: PNTD-D-20-00961_review.pdf [file pntd.0009131.s001.pdf]

Methods

-Are the objectives of the study clearly articulated with a clear testable hypothesis stated?
-Is the study design appropriate to address the stated objectives?
-Is the population clearly described and appropriate for the hypothesis being tested?
-Is the sample size sufficient to ensure adequate power to address the hypothesis being tested?
-Were correct statistical analysis used to support conclusions?
-Are there concerns about ethical or regulatory requirements being met?

This is a qualitative study. As such, its aim is not to "test" hypotheses but to explore the qualities of a specific phenomenon. The study design is appropriate to explore the aim stated in the Author's summary (although there is a discrepancy in clarity between this and the abstract).

The sample size, as it is often the case with qualitative studies, is small. However, the data produced in the text and discussed by the authors show consistently recurrent themes.

The thematic analysis, discussed in the methods and exemplified in the tables, is easy and clear to follow and it is coherent with the Discussion section.

I have no concern about ethical of regulatory requirements. The authors state that they have used pseudonyms, and I did not find details that could lead to identification of participants.

Results

-Does the analysis presented match the analysis plan?
-Are the results clearly and completely presented?
-Are the figures (Tables, Images) of sufficient quality for clarity?

The analysis presented partially matches the analysis plan. The aim of the manuscript is not stated clearly. In the abstract, the authors mention social, economic, political, cultural impacts of NTDs but, throughout the text, they focus on the impact of social, economic, political, cultural factors “on” NTDs—which, I suspect, is the overall aim. I thus suggest rephrasing the mention in the abstract.

The figures and tables are clear.

Conclusions

-Are the conclusions supported by the data presented?
-Are the limitations of analysis clearly described?

-Do the authors discuss how these data can be helpful to advance our understanding of the topic under study?
-Is public health relevance addressed?

(Limit 20000 Characters)

This manuscript lacks a “Conclusions” section and the authors only add a paragraph at the end of the Discussion. While the authors do state their advocacy for a broader approach to tackling NTDs quite clearly, and the public health relevance is evident, they do not satisfactorily engage with previous works and the limitations of the study are not addressed. For instance, small samples are the norm in qualitative studies, but this should still be acknowledged, as well as the fact that individuals were interviewed (alone or in focus group discussions), which are methods with their own sets of bias.

Editorial and Data Presentation Modifications?

Use this section for editorial suggestions as well as relatively minor modifications of existing data that would enhance clarity. If the only modifications needed are minor and/or editorial, you may wish to recommend “Minor Revision” or “Accept”. (Limit 20000 Characters)

The manuscript needs to be thoroughly proofread. The style and register are inconsistent, there are some conceptual and literal repetitions (see lines 162-163; 231/33 and 240/41) and isolated expressions that do not match the overall tone (174: “jumping off point”; 550: “punish”). The Vancouver style is used without in-text citations (e.g. “as [37] suggests” instead of “as Smith [37] suggests”), which make the reading experience unnecessarily difficult. The Results sections, as reported, are uneven. I suggest merging 1.6 Social Factors with 1.7 Cultural Factors, and renaming 1.8 along the lines of “Water, Sanitation, and Infrastructure”.

Summary and General Comments

Use this section to provide overall comments, discuss strengths/weaknesses of the study, novelty, significance, general execution and scholarship. You may also include additional comments for the author, including concerns about dual publication, research ethics, or publication ethics. If requesting major revision, please articulate the new experiments that are needed. (Limit 20000 Characters)

Descriptive papers highlighting the complexity of persisting diseases are needed, most especially when addressing marginalised groups (often blamed for “poor practices”), and this paper has the merit of bringing together different ‘bottom-up’ perspectives. However, there are a few discrepancies between what the authors claim they will do and what they actually do. The authors mention but do not elaborate on the analytical framework, which

remains in the background without tying the data together. The Discussion follows the different sections of the Results but does not engage with the previous literature on the topic, and one is left wondering what is it that they were supposed to notice but did not. The reader needs the authors to tell them where to look among the data collected and how to look at them.

By contrast, some sentences are both very direct and very confusing, seemingly establishing unidirectional relationships of cause and consequence between factors. For instance, 111-113: "Politics determine how government functions are carried out when power is unevenly distributed; it creates a loophole for the exploitation of the poor, which hinders an egalitarian economy, society and preserves inequalities". Surely politics determines but also results from the uneven distribution of power. It is also unclear if the authors are referring to Kenya or making a general point. While I agree with the spirit of the sentence, it is vague and convoluted, and it would be improved by some examples (other countries, other authors – not simply referenced but brought in the discussion) to help spell out what they are referring to exactly. In lines 168-169, it is not clear who the subject is. Who is competing with whom?

What are the implications of this paper for contexts that are not the counties involved? I am not sure that the notion of 'proxy', mentioned in the Introduction and at the beginning of the Discussion is the most useful. It suggests that one (poverty) can be replaced by the other (NTDs). I am not certain this is the point the authors want to make and the phrasing echoes positivist methods such as regression analysis.

The authors mention the necessity to explore the social, political, economic factors that shape health and wellbeing (160-161). I agree, but the topic is not new; it has been explored – in different contexts, in different manners. Yet, the authors do not engage with such literature and sometimes simply mention it. The paragraph between lines 140-148, for instance, could be rephrased to imply that the experience of stigma is similar everywhere, because all systems sanction morality. The role of stigma is to confine a source of danger (to society's culture, identity, norms etc), thus exclusion is the mechanism through which stigma works. I understand that this is not the focus of this manuscript, but leaving concepts such as stigma and witchcraft un-problematised (especially when other disciplines have abundantly done so) on a manuscript that (I hope) will be widely read by policy/global health/epidemiology experts seems counterproductive to the broader aim of this work—which, if I understood correctly, it to expand our perspective on NTDs. I believe the Introduction and the Discussion should dedicate more space to engage with both existing works and policy implications related to the data the authors present. It will strengthen the conclusion of the paper.
On that note, I suggest a separate section for the Conclusions to which, as it is, only two sentences are dedicated.

Confidential Comments to Editor

Comment here if you have any editorial concerns that should be relayed confidentially to the editor and/or journal staff. Comments entered in this field will NOT be communicated to the authors. (Limit 20000 Characters)
