# Supplementary material for: The impact of Neglected Tropical Diseases (NTDs) on health and wellbeing in sub-Saharan Africa (SSA): A case study of Kenya
Source: PLoS Negl Trop Dis. 2021 Feb 11;15(2):e0009131. doi: 10.1371/journal.pntd.0009131 (PMC7904142; doi:10.1371/journal.pntd.0009131)
Supplement: Attachment — Submitted filename: PLOS NTDs Kenya review Aug 6.docx [file pntd.0009131.s002.docx]

PLOS NTDs Kenya review

This paper uses qualitative methods, in the form of KIIs and focus groups to “identif[y] the political, economic, social, and cultural impacts of NTDs on health and wellbeing” in Kenya.

The authors conduct focus groups and key informant interviews to identify political, economic and social effects of NTDs on health and wellbeing. The authors identify a large number of factors which contribute to the NTD burden in Kenya, as well as ways in which NTDs harm health and well being. Some of the authors’ conclusions are that an equity lens should be used to distribute resources, and that poverty alleviation is a way to tackle NTDs.

I have mixed views on this paper. On the positive side, the topic is important, the paper is well-written, the qualitative fieldwork and data handling seems skillfully done, and the voices of the informants that are presented paint a vivid and detailed picture of the many ways that NTDs harm health and well being in Kenya.

On the negative side, I am not sure what is learned that is new in this paper. The connections between poverty, poor living conditions, inadequate sanitation, and NTDs, have been well known and understood for a very long time. In a sense the paper restates the basic tenets of “social determinants of health” – that economic, social and political conditions shape population health outcomes. I believe that this is very well established, including with respect to NTDs.

Thus the main contributions of the paper might be to highlight some Kenya-specific factors which drive the poverty – NTD relationship, such as challenges to the health sector posted by devolution and issues around political accountability such that political leaders do not have incentives to address NTDs (although on this it might be worth noting that Kenya, unlike several other countries in the region, has implemented large scale school-based NTD programs).

I also believe that there is also at times confusion between what can be established by the qualitative methods: “The study establishes that NTDs affect the economic opportunity and productivity of infected persons.” These qualitative methods are certainly suggestive of links between NTDs and economic productivity but I think the authors could certainly soften their claims.

In a related point, since they make arguments about NTDs and productivity in Kenya they might be interested to read some recent experimental work showing that one form of NTD control – mass drug administration against STH – had large impacts on economic productivity in a large trial in western Kenya (<http://emiguel.econ.berkeley.edu/research/twenty-year-economic-impacts-of-deworming>). Given the strong claims about the deleterious effect of NTDs it might also be worth noting the debate about MDA for STH, with a recent Cochrane review finding no benefit while other find important weight and other nutrition gains. (<https://northwestern.app.box.com/s/lae9yxlicdy1ryt16vg7nn0f29oqer2x>)

Smaller issues:

In introduction, authors state that there has been “rampant increases in infectious disease” in Kenya, cite to 2013-2014 DHS. Does this DHS show “rampant increase?” It may, but my memory of this report was that it showed gradual improvement in health conditions. The authors may be correct and I may be misremembering but it is worth double checking this reference.

P5: “NTDs remain endemic to many regions left behind by socioeconomic progress and cause a heavy burden of disability that exceeds malaria and tuberculosis.” This needs a citation.

P6 : spell out “HAT”

P7 : what does it mean to “to interrogate health discourses” – can this be stated more clearly?
