# Supplementary material for: The impact of Neglected Tropical Diseases (NTDs) on health and wellbeing in sub-Saharan Africa (SSA): A case study of Kenya
Source: PLoS Negl Trop Dis. 2021 Feb 11;15(2):e0009131. doi: 10.1371/journal.pntd.0009131 (PMC7904142; doi:10.1371/journal.pntd.0009131)
Supplement: Attachment — Submitted filename: REVIEWERS COMMENTS AND REVISED RESPONSES 14 OCTOBER 2020.docx [file pntd.0009131.s003.docx]

Reviewer #1

1. The study design is appropriate to explore the aim stated in the Author's summary (although there is a discrepancy in clarity between this and the abstract). The analysis presented partially matches the analysis plan. The aim of the manuscript is not stated clearly. In the abstract, the authors mention social, economic, political, cultural impacts of NTDs but, throughout the text, they focus on the impact of social, economic, political, cultural factors “on” NTDs—which, I suspect, is the overall aim. I thus suggest rephrasing the mention in the abstract.

We have revised the discrepancy in the abstract and the author’s summary to provide a clear aim of the paper.

2. This manuscript lacks a “Conclusions” section and the authors only add a paragraph at the end of the Discussion. While the authors do state their advocacy for a broader approach to tackling NTDs quite clearly, and the public health relevance is evident, they do not satisfactorily engage with previous works and the limitations of the study are not addressed. For instance, small samples are the norm in qualitative studies, but this should still be acknowledged, as well as the fact that individuals were interviewed (alone or in focus group discussions), which are methods with their own sets of bias.

We have broadened the discussion and conclusion as well as discussed the research limitations.

3. The manuscript needs to be thoroughly proofread. The style and register are inconsistent, there are some conceptual and literal repetitions (see lines 162-163; 231/33 and 240/41) and isolated expressions that do not match the overall tone (174: “jumping off point”; 550: “punish”). The Vancouver style is used without in-text citations (e.g. “as [37] suggests” instead of “as Smith [37] suggests”), which make the reading experience unnecessarily difficult. The Results sections, as reported, are uneven. I suggest merging 1.6 Social Factors with 1.7 Cultural Factors, and renaming 1.8 along the lines of “Water, Sanitation, and Infrastructure”.

We have revised the referencing and styles as well as merged the suggested results sections.

4. Descriptive papers highlighting the complexity of persisting diseases are needed, most especially when addressing marginalised groups (often blamed for “poor practices”), and this paper has the merit of bringing together different ‘bottom-up’ perspectives. However, there are a few discrepancies between what the authors claim they will do and what they actually do. The authors mention but do not elaborate on the analytical framework, which remains in the background without tying the data together. The Discussion follows the different sections of the Results but does not engage with the previous literature on the topic, and one is left wondering what is it that they were supposed to notice but did not. The reader needs the authors to tell them where to look among the data collected and how to look at them.
   By contrast, some sentences are both very direct and very confusing, seemingly establishing unidirectional relationships of cause and consequence between factors. For

instance, 111-113: “Politics determine how government functions are carried out when power is unevenly distributed; it creates a loophole for the exploitation of the poor, which hinders an egalitarian economy, society and preserves inequalities”. Surely politics determines but also results from the uneven distribution of power. It is also unclear if the authors are referring to Kenya or making a general point. While I agree with the spirit of the sentence, it is vague and convoluted, and it would be improved by some examples (other countries, other authors – not simply referenced but brought in the discussion) to help spell out what they are referring to exactly. In lines 168-169, it is not clear who the subject is. Who is competing with whom?

We have revised the theoretical framework section to make it clear and broadened the discussion section.

5. What are the implications of this paper for contexts that are not the counties involved? I am not sure that the notion of ‘proxy’, mentioned in the Introduction and at the beginning of the Discussion is the most useful. It suggests that one (poverty) can be replaced by the other (NTDs). I am not certain this is the point the authors want to make and the phrasing echoes positivist methods such as regression analysis.
   The authors mention the necessity to explore the social, political, economic factors that shape health and wellbeing (160-161). I agree, but the topic is not new; it has been explored – in different contexts, in different manners. Yet, the authors do not engage with such literature and sometimes simply mention it. The paragraph between lines 140-148, for instance, could be rephrased to imply that the experience of stigma is similar everywhere, because all systems sanction morality. The role of stigma is to confine a source of danger (to society’s culture, identity, norms etc), thus exclusion is the mechanism through which stigma works. I understand that this is not the focus of this manuscript, but leaving concepts such as stigma and witchcraft un-problematised (especially when other disciplines have abundantly done so) on a manuscript that (I hope) will be widely read by policy/global health/epidemiology experts seems counterproductive to the broader aim of this work—which,
   if I understood correctly, it to expand our perspective on NTDs. I believe the Introduction and the Discussion should dedicate more space to engage with both existing works and policy implications related to the data the authors present. It will strengthen the conclusion of the paper.
   On that note, I suggest a separate section for the Conclusions to which, as it is, only two sentences are dedicated.

We have broadened the discussion section and included the conclusion.

Reviewer #2

1. The authors do not provide a clear definition for NTDs. While this may be an NTD journal, it may be worthwhile to consider that a small proportion of readers may not be knowledgeable about this field

We have provided a clear definition of NTDs in the introduction.

2. Any study limitations?

We have provided the study’s limitations.

3. Tables 3-5: I see little value in describing the number of key informants/ focus groups per sub-theme. It really adds no value to a qualitative research given that the ficus is to elicit themes, as against quantify the distribution of responses to themes.

There is more value if the authors consider including subthemes (with narratives and supporting quotes) under each of the   broad themes. For example under Political Factors (which is a deductive theme from the theoretical framework), I'd love to see the emerging themes (such as Devolution of government services and Lack of political will) described in better details with their supporting quotes where necessary.

This is the best way to prove the statement by the Authors under methods section (Lines 241-243) that "Deductive codes captured themes that corresponded to the interview questions, existing literature, and concepts, while inductive codes considered issues emerging from the transcripts"

We appreciate the reviewer's suggestion with respect to the presentation of the qualitative themes.  However, our experience in presenting qualitative research evidence in the global health literature indicates that the use of tables provides a very useful way to visualize the relative importance (as opposed to absolute) of the relevant themes, and is accessible to a proud audience.   We are also supported in this decision by the comments of the other reviewers.

4. Lines 111 to 148 seem to just jump in from no where. There's a need to describe the theoretical basis for considering these factors as important. I suggest that these paragraphs are best  placed under the "Theoretical Framework

We have moved the sentences to the theoretical framework section

5. Theoretical framework: This section should focus on describing the components of the political ecology of health and how this applies to the study design. Lines 162-175 may not be really necessary. The authors should consider replacing this with the content of lines 111-148.

I believe this is a very important and interesting study. Once the authors are able to address these few concerns, the article will be stronger and more appealing to a broader readership.

We have revised the theoretical framework section accordingly.

Reviewer #3

1. This paper uses qualitative methods, in the form of KIIs and focus groups to “identif[y] the political, economic, social, and cultural impacts of NTDs on health and wellbeing” in Kenya.

The authors conduct focus groups and key informant interviews to identify political, economic and social effects of NTDs on health and wellbeing. The authors identify a large number of factors which contribute to the NTD burden in Kenya, as well as ways in which NTDs harm health and well being. Some of the authors’ conclusions are that an equity lens should be used to distribute resources, and that poverty alleviation is a way to tackle NTDs.

I have mixed views on this paper. On the positive side, the topic is important, the paper is well-written, the qualitative fieldwork and data handling seems skillfully done, and the voices of the informants that are presented paint a vivid and detailed picture of the many ways that NTDs harm health and well being in Kenya.

On the negative side, I am not sure what is learned that is new in this paper. The connections between poverty, poor living conditions, inadequate sanitation, and NTDs, have been well known and understood for a very long time. In a sense the paper restates the basic tenets of “social determinants of health” – that economic, social and political conditions shape population health outcomes. I believe that this is very well established, including with respect to NTDs.

Thus the main contributions of the paper might be to highlight some Kenya-specific factors which drive the poverty – NTD relationship, such as challenges to the health sector posted by devolution and issues around political accountability such that political leaders do not have incentives to address NTDs (although on this it might be worth noting that Kenya, unlike several other countries in the region, has implemented large scale school-based NTD programs).

We have revised the discussion sections and highlighted the changes

2. I also believe that there is also at times confusion between what can be established by the qualitative methods: “The study establishes that NTDs affect the economic opportunity and productivity of infected persons.” These qualitative methods are certainly suggestive of links between NTDs and economic productivity but I think the authors could certainly soften their claims.

We have softened the claims in the discussion section

3. In a related point, since they make arguments about NTDs and productivity in Kenya they might be interested to read some recent experimental work showing that one form of NTD control – mass drug administration against STH – had large impacts on economic productivity in a large trial in western Kenya (<http://emiguel.econ.berkeley.edu/research/twenty-year-economic-impacts-of-deworming>). Given the strong claims about the deleterious effect of NTDs it might also be worth noting the debate about MDA for STH, with a recent Cochrane review finding no benefit while other find important weight and other nutrition gains. (<https://northwestern.app.box.com/s/lae9yxlicdy1ryt16vg7nn0f29oqer2x>)

We have discussed the MDA studies in Kenya

Smaller issues:

4. In introduction, authors state that there has been “rampant increases in infectious disease” in Kenya, cite to 2013-2014 DHS. Does this DHS show “rampant increase?” It may, but my memory of this report was that it showed gradual improvement in health conditions. The authors may be correct and I may be misremembering but it is worth double checking this reference.

We have revised the claim accordingly.

5. P5: “NTDs remain endemic to many regions left behind by socioeconomic progress and cause a heavy burden of disability that exceeds malaria and tuberculosis.” This needs a citation.

We have added a citation

6. P6 : spell out “HAT”

We have spelled out the term.

7. P7 : what does it mean to “to interrogate health discourses” – can this be stated more clearly?

We have simplified the sentence structure to make it comprehensive
