# Supplementary material for: The impact of Neglected Tropical Diseases (NTDs) on health and wellbeing in sub-Saharan Africa (SSA): A case study of Kenya
Source: PLoS Negl Trop Dis. 2021 Feb 11;15(2):e0009131. doi: 10.1371/journal.pntd.0009131 (PMC7904142; doi:10.1371/journal.pntd.0009131)
Supplement: Attachment — Submitted filename: Detailed List of Reviewers Comments.docx [file pntd.0009131.s004.docx]

"The Impact of Neglected Tropical Diseases (NTDs) on Health and Wellbeing in sub-Saharan Africa (SSA): A Case Study of Kenya"

550-557: this is possibly the whole point of the paper, which shows that a specific phenomenon (devolution) in a specific context (Kenya) has ignored the issue of NDTs contributing to their exacerbation. Its importance, however, seems to be diluted throughout the paper in favour of more general statements about NDTs and structural poverty. The paper would benefit enormously from having this preceded by a clear statement in the Introduction (e.g. "This paper focuses on post-devolution Kenya to show the political economy of policy initiatives and its influence on NDTs prevalence"). One or two sentences would help us frame the whole paper and the information therein, giving us a straight line to follow the data.

We have made the requested revision.

Always on Table 4 (and this *is* a suggested revision): it is unclear whether the ‘factors’ are listed as promoters or consequences of NDTs. Although references to it are made in the text below, it’d be useful if the authors could add a clearer description.

We have made the requested revision on Table 4 to make it clear that it is both a promoter and a consequence.

Reviewer #1: I think the conclusions could be more precise. The paper presents an interesting case study that has important implications for policy but limits itself to suggesting an 'equity lens' and 'empowerment' to reduce the burden of NDTs. Drawing on the data discussed, I think the authors could suggest more specific ways of using policy towards this aim.

We have made the conclusion more precise.

Reviewer #1: This paper needs to be proofread and consistently formatted. The grammar is OK, but the overall text could be set out in a much more compelling way.

We have proofread and formatted the paper.

Reviewer #1: It feels like the authors want to use one specific context to make broad recommendations, which is not unusual. This aspect of the paper can be found in different sections, but it is never too explicit (until lines 550-557), whereas I think it should be the beacon driving the whole writing. Generally, the writing should be a bit tighter

We have revised the paper accordingly.

34-36: Please replace semi-colons with commas

We have corrected this error.

45-46: The meaning of the sentence is unclear. Perhaps the comma between ‘goal’ and ‘shaping’ is a typo.

We have revised this sentence and added the word “avenue”.

56: Maybe ‘propagate’ is not the best choice. Worsen? Contribute?

We have revised this sentence to “contribute”.

58: ‘as the economies of *some such countries’?

We have revised the sentence to provide more clarity.

61: saying that NDTs area markers for poverty means equalling the two. NDTs are often found in environments characterised by poverty, and the relationship of exacerbation is certainly mutual, but they are not markers nor proxies.

We have clarified the statement.
62: this paper is not a theoretical contribution to knowledge — which is absolutely fine. However, on line 62, I invite the author to rephrase what the paper does. It might be an empirical investigation into X, Y, Z using a political economy approach, for instance, but it does not to ‘extend’ social theory.

We have revised the statement and removed the phrase ‘extend’ social theory.

79-80: ‘shift… shifting’. Maybe a synonym can be found to avoid repetition.

We have changed the word to “shifting”.

98: NDTs contribute to poverty? The verb ‘to promote’ recalls a form of agency that is not part of the theoretical background of this paper (I think).

We have changed the word to “advance”.

207: is there any reason why ‘Namgis’ is between inverted commas? If it is the name of an ethnic group, as suggested by the text, the inverted commas should be removed.

We have removed the inverted commas.

208-210: the description of the study does not appear to be meaningful. Defining one’s “health and wellbeing in terms of political, economic, social, and cultural aspects of their lives and the ease with which they access natural resources” is a phenomenon common to all social groups.

211-212: Maybe these could be moved at the beginning of the explanation?

We have moved the statement to the beginning of the explanation to make it clearer also in our paper we define wellbeing as what makes for a good life.


240: ‘Silenced voices’: I find this expression to be a bit problematic. I understand it is a quotation, but it seems to me that qualitative researchers (or most of them) have moved past the idea that they give voice to the silenced (also, by whom? The passive form leaves us guessing for a subject). So, while I agree that qualitative methods offer a deeper and nuanced understanding of phenomena, my suggestion is to avoid opening the ‘can worm’ of the researcher-participant power relationship. Also, I think the reference is incomplete.

We have removed the phrase “silenced voices”.

395-397: “The results indicate that people infected with NTDs are unable to take part in communal events due to poverty, shame, and information distortion, which makes them marginalized in social affairs”. It is unclear how poverty related to shame and information distortion, here — and it does, but poverty is a phenomenon much broader than shame (i.e. easier to see how shame would directly relate to NDTs).

We have revised the sentence and omitted the word “poverty”.

517: please beware punctuation. Maybe “that NTDs… for example, hydrocele is inherited” could be a viable option?

We have revised the sentence as suggested.

529: the results do not really *demonstrate as in *show.

We have replaced the word demonstrate with “show”.

539-542: that ‘prioritizing political initiatives […] propagates NDTs’ is factually incorrect, as it depends on the kind of political initiatives (as the authors acknowledge towards the end of the sentence). Please rephrase for accuracy.
Also, as above, the verb ‘to propagate’ here might have connotations that are too direct and too unique to be applied to the relationship that X has on NDTs.

We have revised the sentence accordingly.

550-557: this is possibly the whole point of the paper, which shows that a specific phenomenon (devolution) in a specific context (Kenya) has ignored the issue of NDTs contributing to their exacerbation. Its importance, however, seems to be diluted throughout the paper in favour of more general statements about NDTs and structural poverty. The paper would benefit enormously from having this preceded by a clear statement in the Introduction (e.g. "This paper focuses on post-devolution Kenya to show the political economy of policy initiatives and its influence on NDTs prevalence"). One or two sentences would help us frame the whole paper and the information therein, giving us a straight line to follow the data.

We have made it clear within the paper.

630-631: ‘Despite our research contributions, our research outlines some limitations as well’. It’s not the research who outlines; it is the research who has/displays some limitations.

We have revised the statement accordingly.

643: the use of which social theory should be clear by now. There is no need to put that between brackets.

We have revised the statement to include “political ecology of health”.


Reviewer #3: In my first review I noted that positive elements of this paper are that the topic is important, the paper is well-written, the qualitative fieldwork and data handling seems skillfully done, and the voices of the informants that are presented paint a vivid and detailed picture of the many ways that NTDs harm health and well being in Kenya.

On the negative side, I was critical because I was unsure about what was learned that is new in this paper. The connections between poverty, poor living conditions, inadequate sanitation, and NTDs, have been well known and understood for a very long time. In a sense the paper restates the basic tenets of “social determinants of health” – that economic, social and political conditions shape population health outcomes. I believe that this is very well established, including with respect to NTDs.

I still feel that the second critique is valid, as the focus of the paper is on establishing that the social determinants of health contribute to NTDs, which is well known. However, I believe that in this revised version the authors have made their contribution clearer, in part by emphasizing not just issues of poverty, but also how political and governance problems lead to NTDs. In addition, the authors have strengthened the discussion of social and cultural contributors to the harms from NTDs, including stigma and the inaccurate views about the provenance of NTDs. As such, I believe the paper is significantly improved.

Lines 149-150: please reword this sentence for clarity

We have revised the sentence accordingly.

Line 567: are political appointees “non state actors?” If they are political appointees they are by definition part of the state apparatus.

We have revised the statement accordingly.

--------------------
